# Association of multimorbidity with working life expectancy among adults aged 50 years and older: Findings from two prospective cohort studies

**DOI:** 10.1177/26335565251331187

**Published:** 2025-04-15

**Authors:** Katriina Heikkilä, Jaana Pentti, Holendro Singh Chungkham, Sakari Suominen, Mika Kivimäki, Paola Zaninotto, Jenni Ervasti, Jussi Vahtera, Sari Stenholm

**Affiliations:** 1 Department of Public Health, 8058 University of Turku and Turku University Hospital, Turku, Finland; 2 Centre for Population Health Research, 8058 University of Turku and Turku University Hospital, Turku, Finland; 3 Finnish Institute for Health and Welfare, Tampere, Finland; 4 Clinicum, Faculty of Medicine, University of Helsinki, Helsinki, Finland; 5 Psychobiology and Epidemiology Division, Department of Psychology, 7675 Stockholm University, Stockholm, Sweden; 6 Finnish Institute of Occupational Health, Helsinki, Finland; 7 Brain Sciences, 4919 University College London, London, United Kingdom; 8 Department of Epidemiology and Public Health, 4919 University College London, London, United Kingdom; 9 Research Services, 8058 Turku University Hospital and University of Turku, Turku, Finland

**Keywords:** multimorbidity, physical-mental multimorbidity, working life expectancy, register data, cohort study

## Abstract

**Background:**

Individual diseases are important risk factors for early exit from the labour force among older adults, but the contribution of multimorbidity to working life expectancy (WLE) is unclear.

**Methods:**

We used data from two prospective cohort studies: Finnish Public Sector study (FPS) and Health and Social Support Study (HeSSup). Multimorbidity at baseline was ascertained from a combination of self-reported, physician-diagnosed chronic diseases, and nationwide cancer and medication reimbursement registers. WLE from age 50 up to 68 years was ascertained utilising linked data from a nationwide register of pensionable earnings. WLE was estimated utilising a multi-state models in R.

**Results:**

Our findings were based on data from 56,079 women and 17,078 men aged ≥50 years. In FPS, women and men with two chronic diseases could expect to work about 9 months less and those with three or more chronic diseases could expect to work about a year less than those with no chronic disease. In HeSSup, women and men with three or more diseases had about 2-3 years shorter WLEs than those with no disease. In both studies participants with physical-mental multimorbidity had 3-12 months shorter WLEs and individuals with multimorbidity comprising two physical diseases had 8-10 months shorter WLEs than those with no chronic disease. The patterns were similar across the socioeconomic positions.

**Conclusion:**

Women and men with multiple chronic diseases could expect to work ∼1 year less than those with no chronic disease. The differences in WLE can have important economic implications to individuals, health services and society.

## Introduction

Population ageing and the old age dependency ratio increasing have led to many countries putting forward policy measures to promote people staying in the work force for longer. These measures include increasing the statutory retirement age,^1–4^ tightening the eligibility criteria for disability pension^2,5^ and limiting the availability of early voluntary retirement.^1,2^

Poor health is the most common reason for leaving working life before the statutory retirement age and must be considered when developing policies aiming to lengthen working lives.^1–3^ People with a chronic disease tend to have fragmented work careers characterised by, for example, spells of unemployment, and they tend to retire earlier (voluntarily or due to disability) than individuals without chronic disease.^6–8^ Particularly cardiovascular disease, diabetes, musculoskeletal disease and long-term mental health conditions present barriers to work participation.^9^ Evidence from a recent review suggest that adults aged 18 to 59 years with depressive symptoms spend more time on sick leave and unemployed than those without depressive symptoms, and that osteoarthritis, cardiovascular diseases and diabetes were linked to an increased risk of early retirement among older workers.^10^ In a similar vein, the findings from a prediction modelling study suggest that the number of chronic diseases and certain multimorbid disease patterns are important predictors of work disability, along with conventional risk factors, such as age, history of sickness absence and health behaviours.^7^

Multimorbidity - the co-occurrence of multiple chronic or long-term diseases or health conditions in one individual - has become a major health and healthcare concern worldwide.^11,12^ Multimorbidity affects a considerable proportion of adults, with an estimated prevalence of 47% (95% CI: 42 to 53) among those aged ≥50 years.^13^ The Organisation for Economic Co-operation and Development estimates suggest that about a half of adults aged 50-59 years with two or more chronic conditions are not in employment.^14^ Adverse health outcomes associated with multimorbidity among working age and older adults include increased healthcare utilisation and decreased quality of life.^15,16^ However, the extent to which multimorbidity impacts on working life expectancy (the number of years a person can expect to work from a given age) among adult workers aged 50 years and older is unclear and we are not aware of previous studies of this topic. Overall multimorbidity and specific disease combinations could have different impacts on people’s ability to work, depending, for instance, on the requirements of specific jobs and the extent to which healthcare systems can support managing co-occurring diseases (e.g. physical illness together with a mental health conditions, which are treated in different healthcare specialities).^17,18^ Working life expectancy is a holistic, comprehensive measure of work participation and investigating this, rather than single exit routes from employment (e.g. disability retirement, unemployment or premature death^10^) provides a comprehensive and tangible picture of working life among older workers. To add to the evidence, we have examined chronic disease multimorbidity as a determinant of working life expectancy among adults aged 50 years and older, utilising register-linked data from two longitudinal cohort studies.

## Methods

### Study population

**Figure 1.**
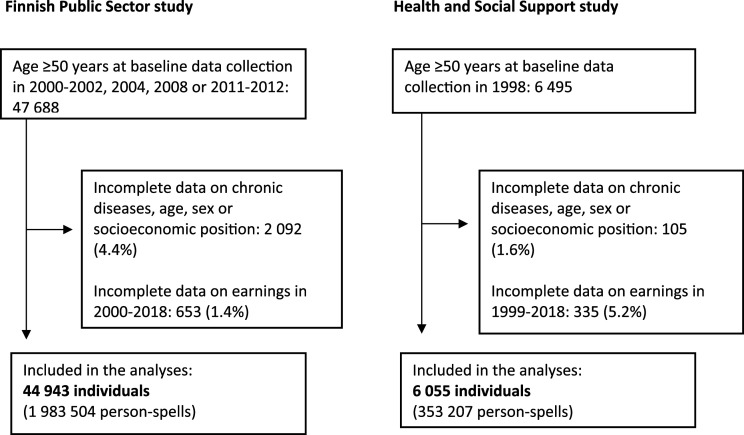
Participant flow chart.

We used data from two prospective cohort studies: the Finnish Public Sector study (FPS) and Health and Social Support study (HeSSup). Full details of the study design, participant recruitment and data collection have been reported previously.^19^ Briefly, FPS, initiated in 2000-2002 is a dynamic occupational cohort of public sector personnel in 10 municipalities and 21 hospitals in Finland.^20^ HeSSup is a population-based cohort study, which was began in 1998 based on a stratified random sample of the Finnish population in four age groups (20–24, 30–34, 40–44, and 50–54 years).^21^ In both studies, data were collected by self-completed questionnaires and the participants’ records were linked to the employers’ registers (FPS) as well as nationwide healthcare registers and population data (both studies) for information on disease diagnoses, medication prescriptions and death. Our analyses in both studies were based on data from participants who were 50 years old or older at analytical baseline (data collection waves 2000-2002, 2004, 2008 or 2012 in FPS and 1998 in HeSSup), had data available on chronic diseases, covariates and working spells in the Earnings and Accrual Register from the baseline up to the end of 2018 (Figure 1). Individuals with missing data on age, sex, socioeconomic position, chronic diseases or pensionable earnings were excluded from the analyses.

### Chronic diseases and multimorbidity at baseline

Chronic diseases and chronic disease multimorbidity were ascertained at analytical baseline, based on self-reports of physician-diagnosed respiratory disease, hypertension, coronary heart disease or stroke, musculoskeletal disorder, migraine, depression, diabetes and cancer; the responses were augmented with data from the nationwide cancer register (to ensure full identification of cancer cases) and medication reimbursement register (to aid identification of respiratory disease, hypertension, coronary heart disease or stroke, musculoskeletal disorder and diabetes; Online Supplement, Supplemental Table 1).^7^ Overall multimorbidity was operationalised as the number of co-occurring diseases: 0, 1, 2 or ≥3. Physical multimorbidity was operationalised as ≥2 physical conditions and physical-mental multimorbidity as ≥1 physical condition together with depression. Specific types of multimorbidity were operationalised as disease combinations of each disease with one other disease or ≥2 other diseases.

### Working life expectancy from age 50 up to 68 years

In our investigation, work participation following the baseline assessment of individual diseases and multimorbidity was ascertained utilising data from Earnings and Accrual Register, maintained by the Finnish Centre for Pensions. The register contains data on all Finnish residents’ earnings (e.g. wages and salaries from employment and self-employed work, as well as social security benefits that are payable to the individual and accrue pension benefits). Data on earnings from employment are recorded from beginning of the month following each individuals’ 17^th^ birthday up to the end of the month of their 68^th^ birthday; data on self-employed earnings are recorded from age 18 up to 68 years.^22^ The register contains information on the dates of beginning and end of each episode of employment. To avoid the fragmentation of the outcome estimates with frequent work status changes resulting from very short spells of work (e.g. a few days of not working between job contracts), we aggregated the data to three-month spells beginning on each individuals’ baseline date. We defined individuals as being at work if they spent any amount of time working during a spell and not working if they spent no time working. Information on dates of death was obtained from Statistics Finland population data. Death was modelled as an additional period beginning on the date of death.

### Covariates

Covariates were factors that previous research suggests are important determinants of multimorbidity and work participation^6,10,19^: sex, age at baseline and follow-up, and socioeconomic position at baseline. Age and sex were ascertained from the employer’s records in FPS and from Statistics Finland population data in HeSSup. Socioeconomic position was ascertained from job titles obtained from the employer’s records in FPS and self-reported level of education in HeSSup. In FPS, data on occupation were converted to International Standard Classification of Occupations (ISCO) by Statistics Finland^23^ and categorised into high (ISCO categories 1-2, e.g. managers or physicians), intermediate (ISCO categories 3-4; skilled non-manual occupations, e.g. registered nurses) and low (ISCO categories 5-9, service and manual occupations, e.g. maintenance workers). In HeSSup, socioeconomic position was categorised as high (university-level qualification), intermediate (occupational college or equivalent) and low (no occupational qualification).

### Statistical methods

Working life expectancy from age 50 up to 68 years was estimated utilising a multi-state life tables approach, based on transition probabilities between three states (working, not working and dead). We used R packages *msm* (for multi-state survival models in panel data^24^) to predict transition probabilities across the three states (Online Supplement, Supplemental Figure 1): individuals could be in one state at a time but move between working and not working, with death modelled as an absorbing state. The msm package cannot incorporate weights and the analyses were not weighted. R package *elect*, utilising a Gombertz model that defines age as a time-dependent covariate,^25^ was used to estimate working life expectancies for individuals with and without overall multimorbidity, specific types of multimorbidity and individual chronic diseases at age ≥50 years.^26^ Age in the beginning of each state was modelled as a covariate. The WLE predictions were obtained separately for men and women, and for socioeconomic position (low, intermediate and high). This was done because previous research indicates that work participation and the duration of working life differ by sex and socioeconomic position.^27,28^ We calculated 95% confidence intervals for the differences in WLEs from 500 repeated simulations based on asymptotic properties of maximum likelihood estimator for the multi-state models. To examine the potential impact of the differences in baseline working status, we undertook a sensitivity analysis in HeSSup restricting the study population to those who were working at baseline. Analyses were conducted using SAS 9.4 (SAS Institute, Cary, North Carolina, US) and R 4.3.2. (R Foundation for Statistical Computing, Vienna, Austria).

## Results

Our analyses were based on data from 44 943 participants from the FPS (80% women) and 6 055 participants from HeSSup (55% women) (Figure 1). The participants were, on average, 52 to 54 years old at baseline. In the FPS all participants were working at baseline, whereas HeSSup also included participants (∼20%) who did not work at baseline (Table 1). Just over a third of the participants had a low socioeconomic position in both studies. FPS included larger proportions of participants in high socioeconomic positions, whereas in HeSSup, the majority of the participants were in the intermediate category.

**Table 1.** Participant characteristics at baseline.

	FPS				HeSSup			
	Men (n=8963)		Women (n=35980)		Men (n=2747)		Women (n=3308)	
	N	%	N	%	N	%	N	%
Age, mean (SD)	54.2 (3.3)		53.7 (3.1)		51.9 (1.4)		51.9 (1.4)	
Range	50 to 67		50 to 67		50 to 54		50 to 54	
Work status								
Working	8963	100	35980	100	2122	77.3	2671	80.7
Not working	0	0	0	0	625	22.8	637	19.3
Socioeconomic position								
High	3876	43.2	10514	29.2	446	16.2	475	14.4
Intermediate	1824	20.4	11442	31.8	1326	48.3	1528	46.2
Low	3263	36.4	14024	39.0	975	35.5	1305	39.5
Disease								
Respiratory disease	1123	12.5	5256	14.6	400	14.6	565	17.1
Hypertension	2508	28.0	8167	22.7	504	18.4	478	14.5
Coronary heart disease or stroke	391	4.4	644	1.8	181	6.6	76	2.3
Musculoskeletal disorder	3390	37.8	15186	42.2	959	34.9	1314	39.7
Migraine	894	10.0	8474	23.6	329	12.0	929	28.1
Depression	1017	11.4	5294	14.7	414	15.1	585	17.7
Diabetes	606	6.8	1257	3.5	151	5.5	121	3.7
Cancer	447	5.0	1697	4.7	73	2.7	220	6.7
Overall multimorbidity								
No disease	3008	33.6	10392	28.9	1016	37.0	977	29.5
1 disease	3123	34.8	12520	34.8	911	33.2	1119	33.8
2 diseases	1757	19.6	7893	21.9	512	18.6	698	21.1
≥3 diseases	1075	12.0	5175	14.4	308	11.2	514	15.5
Physical-mental multimorbidity								
≥2 physical diseases	2026	22.6	8861	24.6	505	18.4	719	21.7
≥1 physical diseases and depression	806	9.0	4207	11.7	315	11.5	493	14.9

Notes: FPS: Finnish Public Sector study, HeSSup: Health and Social Support study.

The most common chronic diseases among the cohort participants were musculoskeletal disorders (affecting over a third of all participants) and hypertension (affecting about a quarter of the participants in FPS and around 15% in HeSSup) (Table 1). Respiratory disease, migraine and depression were reported by 10% to 28% of the participants (with the proportions varying somewhat in men and women and by study). Cancer, diabetes, coronary heart disease and stroke were relatively rare, affecting <7% of the participants. In terms of multimorbidity, most participants had none of the chronic diseases examined in our analyses, about a third had one disease, a fifth had two diseases and 11% to 15% had three or more diseases. Multimorbidity consisting of two or more physical diseases was reported by about a fifth of the participants and physical-mental multimorbidity (i.e. depression with one or more physical diseases) was observed in about 10% of the participants (Table 1). The most common disease combinations making up multimorbidity were depression together with one or more other diseases (4 to 9% of participants overall) and musculoskeletal disorders together with one or more other diseases (9 to 15% of participants overall). Multimorbidity patterns comprising cancer, coronary heart disease or stroke, and diabetes affected 0.4 to 3.5% of participants (Online Supplement, Supplemental Table 2).

Overall, FPS participants could expect to work for 13.01 years (95% CI: 12.98 to 13.04) and Hessup participants could expect to work for 9.59 years (95 % CI: 9.50 to 9.68) from age 50 up to 68 years Generally, individuals who had one or more chronic diseases had shorter WLEs than those who had no chronic disease (Table 2; Online Supplement, Supplemental Tables 3 and 4). Estimated working life expectancies at age 50 up to 68 years among participants who had no chronic disease were 13.47 years (95% CI: 13.43 to 13.52) in FPS and 10.58 years (95% CI: 10.43 to 10.72) in HeSSup (Online Supplement, Supplemental Table 3). In the FPS, women and men with two chronic diseases could expect to work about 9 months less and those with three or more chronic diseases could expect to work about a year less than those with no chronic disease; this pattern was similar across the socioeconomic positions but the estimated working life expectancies were the shortest among participants in the lowest socioeconomic groups (Table 2. In HeSSup, the pattern of a lower working life expectancy among women and men with three or more diseases was even starker, about 2 to 3 years, compared to those with no disease (Table 3). Findings from the sensitivity analyses in HeSSup suggest that in this study, women and men who were working at study baseline could expect to work longer that those who were not working at baseline. The WLE patterns by sex and socioeconomic position in this subgroup were similar to our main findings (Online Appendix, Supplemental Table 4).

**Table 2.** Estimated working life expectancies from age 50 up to 68 years in Finnish Public Sector study, by multimorbidity, sex and socioeconomic position.

	Men																	
	High SEP						Intermediate SEP						Low SEP					
	WLE	95% CI		Months lost	95% CI		WLE	95% CI		Months lost	95% CI		WLE	95% CI		Months lost	95% CI	
Overall multimorbidity																		
No disease	13.87	13.78	13.95	0	-	-	13.53	13.43	13.62	0	-	-	13.11	13.03	13.20	0	-	-
1 disease	13.45	13.36	13.54	4.9	3.5	6.4	13.12	13.02	13.22	5.0	3.3	6.6	12.70	12.61	12.79	5.0	3.6	6.4
2 diseases	13.11	13.01	13.20	9.1	7.6	10.6	12.78	12.66	12.89	9.0	7.2	10.8	12.36	12.25	12.45	9.1	7.6	10.6
≥3 diseases	12.80	12.68	12.91	12.8	11.0	14.5	12.46	12.31	12.58	12.9	10.8	14.9	12.04	11.89	12.17	12.9	11.0	14.8
Physical-mental multimorbidity																		
≥2 physical diseases	13.10	13.00	13.19	9.2	7.8	10.7	12.75	12.65	12.85	9.3	7.6	11.0	12.33	12.23	12.43	9.4	7.9	11.0
≥1 physical diseases and depression	12.78	12.66	12.90	13.1	11.3	14.8	12.44	12.30	12.55	13.1	11.2	14.9	12.01	11.89	12.14	13.2	11.4	15.0

	Women																	
	High SEP						Intermediate SEP						Low SEP					
	WLE	95% CI		Months lost	95% CI		WLE	95% CI		Months lost	95% CI		WLE	95% CI		Months lost	95% CI	
Overall multimorbidity																		
No disease	13.84	13.78	13.90	0	-	-	13.53	13.47	13.59	0	-	-	13.10	13.04	13.16	0	-	-
1 disease	13.44	13.37	13.49	4.8	3.8	5.8	13.13	13.07	13.20	4.8	3.7	5.8	12.70	12.65	12.76	4.8	3.8	5.8
2 diseases	13.09	13.02	13.16	9.0	7.9	10.1	12.79	12.71	12.86	8.9	7.8	10.0	12.36	12.28	12.42	8.9	7.8	10.1
≥3 diseases	12.82	12.72	12.89	12.3	11.0	13.5	12.51	12.42	12.60	12.2	10.9	13.5	12.08	11.99	12.16	12.3	11.0	13.6
Physical-mental multimorbidity																		
≥2 physical diseases	13.10	13.02	13.16	8.9	7.8	10.0	12.79	12.71	12.86	8.9	7.8	10.0	12.35	12.29	12.42	9.0	7.9	10.1
≥1 physical diseases and depression	12.76	12.67	12.85	13.0	11.7	14.3	12.45	12.36	12.54	12.9	11.6	14.2	12.01	11.93	12.10	13.1	11.8	14.3

Notes: WLE: working life expectancy; SEP: socioeconomic position. Months lost: months lost compared to having no disease.

**Table 3.** Estimated working life expectancies from age 50 up to 68 years in Health and Social Support study, by multimorbidity, sex and socioeconomic position.

	Men																	
	High SEP						Intermediate SEP						Low SEP					
	WLE	95% CI		Months lost	95% CI		WLE	95% CI		Months lost	95% CI		WLE	95% CI		Months lost	95% CI	
Overall multimorbidity																		
No disease	12.05	11.78	12.28	0	-	-	10.51	10.31	10.68	0	-	-	9.55	9.31	9.73	0	-	-
1 diasease	11.30	11.01	11.53	8.9	4.6	13.3	9.73	9.53	9.94	9.4	6.2	12.7	8.73	8.50	8.92	9.8	6.2	13.3
2 diseases	10.54	10.19	10.80	18.0	13.3	22.8	8.94	8.67	9.19	18.8	15.0	22.6	7.86	7.57	8.11	20.2	16.0	24.3
≥3 diseases	9.33	8.95	9.68	32.6	27.3	38.0	7.62	7.33	7.86	34.7	30.8	38.5	6.63	6.15	6.91	35.0	29.8	40.2
Physical-mental multimorbidity																		
≥2 physical diseases	10.54	10.21	10.86	18.1	13.2	23.1	8.89	8.64	9.11	19.5	15.9	23.1	7.82	7.54	8.05	20.7	16.8	24.7
≥1 physical diseases and depression	9.50	9.10	9.81	30.6	25.4	35.8	7.76	7.46	8.03	33.1	29.0	37.2	6.69	6.35	6.95	34.3	29.9	38.7

	Women																	
	High SEP						Intermediate SEP						Low SEP					
	WLE	95% CI		Months lost	95% CI		WLE	95% CI		Months lost	95% CI		WLE	95% CI		Months lost	95% CI	
Overall multimorbidity																		
No disease	12.30	12.08	12.56	0	-	-	10.78	10.58	10.97	0	-	-	9.90	9.72	10.12	0	-	-
1 diasease	11.58	11.32	11.82	8.7	4.5	12.8	10.01	9.83	10.19	9.2	6.0	12.4	9.11	8.93	9.29	9.5	6.2	12.7
2 diseases	10.88	10.62	11.13	17.1	12.8	21.3	9.28	9.06	9.51	18.0	14.5	21.6	8.35	8.11	8.56	18.7	15.0	22.3
≥3 diseases	9.64	9.31	9.96	31.9	27.1	36.8	7.96	7.71	8.24	33.9	29.9	37.8	7.01	6.76	7.25	34.7	30.9	38.5
Physical-mental multimorbidity																		
≥2 physical diseases	10.84	10.56	11.12	17.5	13.1	21.9	9.20	8.96	9.42	19.0	15.4	22.6	8.25	8.04	8.45	19.8	16.3	23.3
≥1 physical diseases and depression	9.82	9.46	10.18	29.8	24.6	35.0	8.10	7.83	8.34	32.2	28.3	36.1	7.11	6.84	7.37	33.5	29.5	37.5

Notes: WLE: working life expectancy; SEP: socioeconomic position. Months lost: months lost compared to having no disease.

In both studies participants with physical-mental multimorbidity had from about 3 months to 1 year shorter working life expectancies than those with no chronic disease; individuals with multimorbidity comprising two physical diseases could expect to work about 8 to 10 months less than those with no chronic disease (Tables 2 and 3). Individuals with multimorbid disease combinations consisting of coronary heart disease or stroke, or diabetes with ≥2 other diseases had the shortest WLEs when compared to individuals with no disease (Online Supplement, Supplemental Tables 5 and 6).

Of the individual chronic diseases, the estimated working life expectancies were the shortest among participants with cancer or coronary heart disease or stroke in the FPS and among those with coronary heart disease or stroke, depression or diabetes in HeSSup (Online Appendix, Supplemental Table 7). Diseases that were the least strongly associated with working life expectancy were migraine and musculoskeletal diseases (in both studies), hypertension (in FPS) and cancer (in HeSSup).

## Discussion

Multimorbidity poses and significant challenge to policies aiming to extend working lives; appropriate provision of healthcare and the management of chronic diseases will have a key role in encouraging and enabling people to work up to or even beyond their statutory pension age. Our findings suggest that the socioeconomic gradient seen in many health outcomes, including multimorbidity, is also evident in their working life consequences. Our observations suggest that people with chronic disease multimorbidity at age 50 up to 68 years could expect to work for some 2 to 3 years less than those with no chronic disease. Overall, the patterns of the predicted working life expectancies were similar in men and women but differed by socioeconomic position: individuals in higher socioeconomic positions could expect to work longer, with shorter working life expectancies (up to ∼9 months) predicted for women and men in lower socioeconomic positions. Our observations point to a dose-response relationship between an increasing complexity of multimorbidity and a shorter working life expectancy.

We used data from two independent cohort studies to provide a comprehensive picture of the association of multimorbidity with working life expectancy among older adults. Overall, the study population in FPS, consisting of public sector employees, contains larger proportions of women and participants with high socioeconomic position than the study population in HeSSup, which is based on a population sample. This may explain the overall longer WLEs and less stark socioeconomic differences in WLE in FPS compared to HeSSup. One interpretation for these findings could be that the public sector employees in FPS have access to occupational healthcare provided by the employer, whereas HeSSup includes workers in other sectors, some of whom may have more limited occupational healthcare provision and rely solely or to a larger extent on public healthcare, which is available to all in Finland. Whilst the universal healthcare system in the country overall has good coverage and service, occupational healthcare plans tend to have shorter waiting times for initial appointments and may offer easier access to specialised assessment, which may impact on workers’ ability to manage multiple conditions and continue to work with these. Also, HeSSup contains a larger proportion of participants who were not working at baseline, which may explain the overall shorter working life expectancies in this study population, as individuals with a chronic disease who do not work are less likely to start or resume working that those with no chronic disease.^18^ This is supported by the findings from the sensitivity analyses restricting the HeSSup study population to those who were working at study baseline, which pointed to a longer working life expectancy in this group in comparison to the overall HeSSup population.

The working life expectancies among FPS and HeSSup participants reflect variation in the prevalence of individual chronic diseases and multimorbidity and the impact these have on people’s work capacity. Our findings suggest that individuals with multiple diseases consistently have shorter working life expectancies than those with any single disease. In both studies the working life expectancies were the shortest among individuals with coronary heart disease or stroke and diabetes with two or more other diseases, which could point to complex multimorbidity with a cardiometabolic component having a particularly detrimental impact on people’s ability to work (e.g. due to the physical or cognitive corollaries of these diseases) or the contribution of these disease combinations in reducing the overall life expectancy among people aged 50 years and older. In both FPS and HeSSup individuals with physical-mental multimorbidity, comprising depression and at least one physical disease, had shorter working life expectancies than those with no disease or those with two or more physical diseases. These observations are in line with those of previous investigations, which suggest that mental health conditions are important determinants of healthy working life expectancy among middle-aged and older adults.^29,30^

### Strengths and limitations

We are not aware of previous studies on chronic disease multimorbidity and working life expectancy. We used prospectively collected data from two cohort studies with up to 18 years of register follow-up; consequently, our findings are unlikely to have been affected by reporting biases, e.g. differential recording or recall of work history among individuals with and without chronic disease. Work participation, used to estimate working life expectancy, was ascertained from nationwide register data on pensionable income. There is no universally agreed, gold standard method for ascertaining working life expectancy. Observational studies have done this utilising self-reported data on working status, collected across multiple data collection waves. Limitations with this approach include potential biases, missing data and low level of detail, as self-reported work status can be influenced by reporting biases, some participants will be lost to follow-up and their work status between the data collection waves it not always known. Our approach, ascertaining working life expectancy based on administrative data on pensionable income, is an attempt to overcome these issues by making use of a unique source of nationwide set of detailed data on daily work participation across the entire study follow-up period. These data comprise near-complete information on participating in paid work during the study follow-up and up to age 68 years, with the exception of work that does not accrue pension or social security benefits in Finland, e.g. working for an employer based outside of the country. The register data also do not include information on grant-funded research or artistic work, and we were thus unable to explore the roles of these types of work on the study participants’ working life expectancy. Although we had no data available on these types of work, we expect them to have been rare in our study populations, with negligible impact on our findings. In a similar vein, the register data do not distinguish certain short spells of sickness absence from working, or whether the participants worked full-time or part-time. As focus of our investigation was on the number of years people could expect to participate in the labour market, our findings do not reflect the contribution of voluntary work, cash-in-hand work or unpaid household or care work to older adults’ overall workload.

Multimorbidity was operationalised as combinations of diseases from a pre-defined list of chronic or long-term diseases and conditions, and consequently does not comprise all disease combinations prevalent among middle-aged and older working adults. Further research would help to elucidate the roles of specific multimorbidity patterns, temporal sequences of diseases and years lived with multimorbidity as predictors of working life expectancy. We excluded from our analyses individuals with missing data on age, sex, socioeconomic position, chronic diseases or pensionable earnings, which are likely to mainly represent data errors. It is possible that these exclusions have diluted some of the estimated associations of multimorbidity with working life expectancy.

Differences in WLE can have important health and economic implications for individuals, ageing populations and society. For example, work participation impacts on access to occupational health care and insurance in settings where these are provided by the employer or in connection with employment, as well as the pension income workers can expect to receive upon retirement. Financial remuneration and benefits aside, work participation is also an important determinant of psychosocial well-being for many people. However, we feel ill-equipped to make specific policy or care recommendations to employers, healthcare providers or policy makers based on findings in two cohort studies in one country. Comparative research in diverse occupational and healthcare settings would help gauge on how workers with single chronic diseases or multimorbidity would be best supported to work in a way that is healthy, productive and meaningful.

### Conclusion

Our findings in two prospective cohort studies in Finland suggest that women and men aged 50 years and older with multiple chronic diseases can expect to work ∼1 year less than those with no chronic disease. The differences in WLE can have important health and economic implications for individuals, ageing populations and society.

## Supplemental Material

Supplemental Material - Association of multimorbidity with working life expectancy among adults aged 50 years and older: Findings from two prospective cohort studies Supplemental Material for Association of multimorbidity with working life expectancy among adults aged 50 years and older: Findings from two prospective cohort studies by Katriina Heikkilä, Jaana Pentti, Holendro Singh Chungkham, Sakari Suominen, Mika Kivimäki, Paola Zaninotto, Jenni Ervasti, Jussi Vahtera, and Sari Stenholm in Journal of Multimorbidity and Comorbidity

## Data Availability

In the Finnish Public Sector Study and Health and Social Support Study, the pseudonymised questionnaire data used in this study can be made available upon request to the study team. Linked data on deaths, specialised healthcare, medication purchases and reimbursement, and pensionable earnings require separate permissions from the Finnish Institute of Health and Welfare, Statistics Finland and the Finnish Centre for Pensions.*

## References

[1] van der Noordt M van der Pas S van Tilburg TG , et al. Changes in working life expectancy with disability in the Netherlands, 1992-2016. Scand J Work Environ Health 2019; 45: 73-81. DOI: 10.5271/sjweh.3765. 30176168

[2] van der Mark-Reeuwijk KG Weggemans RM Bultmann U , et al. Health and prolonging working lives: an advisory report of the Health Council of The Netherlands. Scand J Work Environ Health 2019; 45: 514-519. DOI: 10.5271/sjweh.3828. 31069395

[3] Parker M Bucknall M Jagger C , et al. Population-based estimates of healthy working life expectancy in England at age 50 years: analysis of data from the English Longitudinal Study of Ageing. Lancet Public Health 2020; 5: e395-e403. DOI: 10.1016/S2468-2667(20)30114-6. 32619541

[4] de Wind A van der Noordt M Deeg DJH , et al. Working life expectancy in good and poor self-perceived health among Dutch workers aged 55-65 years with a chronic disease over the period 1992-2016. Occup Environ Med 2018; 75: 792-797. DOI: 10.1136/oemed-2018-105243. 30194272

[5] Kadefors R Nilsson K Ostergren PO , et al. Social inequality in working life expectancy in Sweden. Z Gerontol Geriatr 2019; 52: 52-61. DOI: 10.1007/s00391-018-01474-3. 30413944 PMC6373384

[6] Edge CE Cooper AM Coffey M . Barriers and facilitators to extended working lives in Europe: a gender focus. Public Health Rev 2017; 38: 2. DOI: 10.1186/s40985-017-0053-8. 29450074 PMC5810036

[7] Nyberg ST Airaksinen J Pentti J , et al. Predicting work disability among people with chronic conditions: a prospective cohort study. Sci Rep 2023; 13: 6334. DOI: 10.1038/s41598-023-33120-3. 37072462 PMC10113323

[8] Shiri R Falah-Hassani K Lallukka T . Body mass index and the risk of disability retirement: a systematic review and meta-analysis. Occup Environ Med 2020; 77: 48-55. DOI: 10.1136/oemed-2019-105876. 31467042

[9] de Boer A Geuskens GA Bültmann U , et al. Employment status transitions in employees with and without chronic disease in the Netherlands. Int J Public Health 2018; 63: 713-722. DOI: 10.1007/s00038-018-1120-8. 29846767 PMC6015601

[10] Shiri R Hiilamo A Lallukka T . Indicators and determinants of the years of working life lost: a narrative review. Scand J Public Health 2021; 49: 666-674. DOI: 10.1177/1403494821993669. 33645306 PMC8512267

[11] Ho IS Azcoaga-Lorenzo A Akbari A , et al. Examining variation in the measurement of multimorbidity in research: a systematic review of 566 studies. Lancet Public Health 2021; 6: e587–e597. DOI: 10.1016/S2468-2667(21)00107-9. 34166630

[12] Xu X Mishra GD Jones M . Evidence on multimorbidity from definition to intervention: An overview of systematic reviews. Ageing Res Rev 2017; 37: 53–68. DOI: 10.1016/j.arr.2017.05.003. 28511964

[13] Chowdhury SR Chandra Das D Sunna TC , et al. Global and regional prevalence of multimorbidity in the adult population in community settings: a systematic review and meta-analysis. EClinicalMedicine 2023; 57: 101860. DOI: 10.1016/j.eclinm.2023.101860. 36864977 PMC9971315

[14] OECD . The labour market impacts of ill-health. 2016.

[15] France EF Wyke S Gunn JM , et al. Multimorbidity in primary care: a systematic review of prospective cohort studies. Br J Gen Pract 2012; 62: e297–307. DOI: 10.3399/bjgp12X636146. 22520918 PMC3310037

[16] Pati S Swain S Hussain MA , et al. Prevalence and outcomes of multimorbidity in South Asia: a systematic review. BMJ Open 2015; 5: e007235. DOI: 10.1136/bmjopen-2014-007235. PMC4606435 26446164

[17] Pizzol D Trott M Butler L , et al. Relationship between severe mental illness and physical multimorbidity: a meta-analysis and call for action. BMJ Ment Health 2023; 26: 1. DOI: 10.1136/bmjment-2023-300870. PMC10619039 37907331

[18] Vooijs M Leensen MC Hoving JL , et al. Disease-generic factors of work participation of workers with a chronic disease: a systematic review. Int Arch Occup Environ Health 2015; 88: 1015-1029. DOI: 10.1007/s00420-015-1025-2. 25712761 PMC4608993

[19] Kivimaki M Batty GD Pentti J , et al. Association between socioeconomic status and the development of mental and physical health conditions in adulthood: a multi-cohort study. Lancet Public Health 2020; 5: e140-e149. DOI: 10.1016/S2468-2667(19)30248-8. 32007134

[20] Kivimaki M Lawlor DA Davey Smith G , et al. Socioeconomic position, co-occurrence of behavior-related risk factors, and coronary heart disease: the Finnish Public Sector study. Am J Public Health 2007; 97: 874-879. DOI: 10.2105/AJPH.2005.078691. 17395837 PMC1854863

[21] Korkeila K Suominen S Ahvenainen J , et al. Non-response and related factors in a nation-wide health survey. European journal of epidemiology 2001; 17: 991–999. 12380710 10.1023/a:1020016922473

[22] Finnish Center for Pensions . Earnings and Accrual Register. https://www.etk.fi/en/services-for-experts/joint-registers-and-information-system-services/register-descriptions/ (2024, accessed 18 March 2024 2024).

[23] International Labour Organization . International Standard Classification of Occupations (ISCO-08). International Labour Organisation.

[24] Jackson C . Multi-State Models for Panel Data: The msm Package for R. Journal of Statistical Software 2011; 38: 1–28. 10.18637/jss.v038.i08

[25] van den Hout A Sum Chan M Matthews F . Estimation of life expectancies using continuous-time multi-state models. Comput Methods Programs Biomed 2019; 178: 11-18. DOI: 10.1016/j.cmpb.2019.06.004. 31416539

[26] Chungkham HS Högnäs RS Head J , et al. Estimating Working Life Expectancy: A Comparison of Multistate Models. Sage Open. 2023; 13. 10.1177/21582440231177270

[27] Virtanen M Oksanen T Pentti J , et al. Occupational class and working beyond the retirement age: a cohort study. Scand J Work Environ Health 2017; 43: 426-435. DOI: 10.5271/sjweh.3645. 28504807

[28] Myllyntausta S Virtanen M Pentti J , et al. Why do men extend their employment beyond pensionable age more often than women? a cohort study. European Journal of Ageing 2022; 19: 599–608. DOI: 10.1007/s10433-021-00663-1. 36052186 PMC9424425

[29] Laaksonen M Elovainio M Kainulainen S , et al. Changes in healthy and unhealthy working life expectancies among older working-age people in Finland, 2000–2017. European Journal of Public Health 2022; 32: 729–734. DOI: 10.1093/eurpub/ckac119. 36069835 PMC9527978

[30] Lynch M Bucknall M Jagger C , et al. Demographic, health, physical activity, and workplace factors are associated with lower healthy working life expectancy and life expectancy at age 50. Scientific Reports 2024; 14: 5936. DOI: 10.1038/s41598-024-53095-z. 38467680 PMC10928117

