# Supplementary material for: Association of multimorbidity with working life expectancy among adults aged 50 years and older: Findings from two prospective cohort studies
Source: J Multimorb Comorb. 2025 Apr 15;15:26335565251331187. doi: 10.1177/26335565251331187 (PMC12033512; doi:10.1177/26335565251331187)
Supplement: Supplemental Material - Association of multimorbidity with working life expectancy among adults aged 50 years and older: Findings from two prospective cohort studies [file sj-pdf-1-cob-10.1177_26335565251331187.pdf]

Online supplement

Supplementary figure 1. Three-state model for estimating working life expectancy

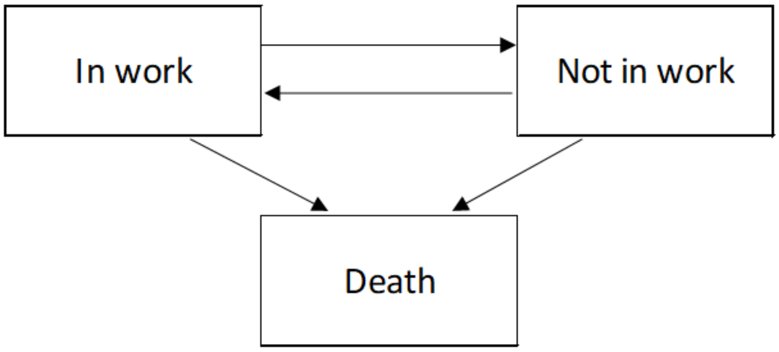

Supplementary table 1. Ascertainment of multimorbidity

Physician-diagnosed disease (self-report)	Electronic health records codes	Multimorbidity outcome
Allergic rhinitis	ICD-10: E84.0, J41- J45<br>Medication reimbursement code: 203 (chronic asthma and other chronic lung diseases)	Respiratory disease
Asthma		
Chronic lung disease		
Hypertension	ICD-10 codes: I10–I13, I15, I27.0, I27.2<br>Medication reimbursement code: 205 (chronic hypertension)	Hypertension
Coronary infarction or embolism	ICD-10: I20–I22, I25<br>Medication reimbursement code: 206 (medical treatment for chronic coronary artery disease)	Coronary heart disease or stroke
Angina pectoris		
Stroke		
Osteoarthritis	ICD-10: M02, M05, M06, M08, M13, M30–M35, M45, M46.1, M46.9, M86.3, M94.1<br>Medication reimbursement code: 202 (chronic rheumatoid arthritis)	Musculoskeletal disorder
Rheumatoid arthritis		
Sciatica		
Migraine	-	Migraine
Depression	-	Depression
Diabetes	ICD-10: E10-E14, E89.1<br>Medication reimbursement code: 103 (insulin for diabetes)	Diabetes
Cancer	ICD-10 codes: C00-C99	Cancer

Note to table: ICD-10: International Classification of Diseases version 10. Medication reimbursement codes are administrative codes used in the Medication Reimbursement Register maintained by the Social Insurance Institution of Finland. The ICD-10 codes in the table indicate the specialist diagnosis needed to be entitled to cost reimbursement for medication for a disease or condition. The reimbursement codes indicate the right to cost reimbursement and are specific to a disease, rather than a medication.

Supplementary table 2. Types of multimorbidity

	FPS				HeSSup			
	Men (n=8963)		Women (n=35980)		Men (n=2747)		Women (n=3308)	
	N	%	N	%	N	%	N	%
**Physical and mental multimorbidity,** %								
≥2 physical diseases	2026	22.6	8861	24.6	505	18.4	719	21.7
≥1 physical diseases and depression	806	9.0	4207	11.7	315	11.5	493	14.9
**Coronary heart disease or stroke and cardiovascular multimorbidity, %**								
Coronary heart disease or stroke only	35	0.4	53	0.2	25	0.9	4	0.1
Coronary heart disease or stroke with 1 other disease	130	1.5	156	0.4	58	2.1	14	0.4
Coronary heart disease or stroke with ≥2 other diseases	226	2.5	435	1.2	98	3.6	58	1.8
**Multimorbidity excluding coronary heart disease or stroke, %**								
2 diseases without coronary heart disease or stroke	1627	18.2	7737	21.5	454	16.5	684	20.7
≥3 diseases without coronary heart disease or stroke	849	9.5	4740	13.2	210	7.6	456	13.8
**Depression and physical-mental multimorbidity, %**								
Depression only	211	2.4	1087	3.0	99	3.6	92	2.8
Depression with 1 other disease	327	3.7	1719	4.8	149	5.4	192	5.8
Depression with ≥2 other diseases	479	5.3	2488	6.9	166	6.0	301	9.1
**Multimorbidity excluding depression, %**								
2 diseases without depression	1430	16.0	6174	17.2	363	13.2	506	15.3
≥3 diseases without depresssion	596	6.7	2687	7.5	142	5.2	213	6.4
**Cancer and malignant multimorbidity, %**								
Cancer only	88	1.0	436	1.2	24	0.9	77	2.3
Cancer with 1 other disease	150	1.7	552	1.5	24	0.9	64	1.9
Cancer with ≥2 other diseases	209	2.3	709	2.0	25	0.9	79	2.4
**Multimorbidity excluding cancer, %**								
2 diseases without cancer	1607	17.9	7341	20.4	488	17.8	634	19.2
≥3 diseases without cancer	866	9.7	4466	12.4	283	10.3	435	13.2
**Diabetes and diabetic multimorbidity, %**								
Diabetes only	87	1.0	170	0.5	28	1.0	20	0.6
Diabetes with 1 other disease	202	2.3	369	1.0	52	1.9	27	0.8
Diabetes with ≥2 other diseases	317	3.5	718	2.0	71	2.6	74	2.2
**Multimorbidity excluding diabetes, %**								
2 diseases without diabetes	1555	17.4	7524	20.9	460	16.8	671	20.3
≥3 diseases without diabetes	758	8.5	4457	12.4	237	8.6	440	13.3
**Musculoskeletal multimorbidity, %**								
Musculoskeletal disorder only	1414	15.8	5447	15.1	396	14.4	442	13.4
Musculoskeletal disorder with 1 other disease	1121	12.5	5310	14.8	312	11.4	450	13.6
Musculoskeletal disorder with ≥2 other diseases	855	9.5	4429	12.3	251	9.1	422	12.8
**Multimorbidity excluding musculoskeletal disorder, %**								
2 diseases without msculoskeletal disorder	636	7.1	2583	7.2	200	7.3	248	7.5
≥3 diseases without musculoskeletal disorder	220	2.5	746	2.1	57	2.1	92	2.8

Note: FPS: Finnish Public Sector study, HeSSup: Health and Social Support study.

Supplementary table 3. Estimated working life expectancies from age 50 up to 68 years, by multimorbidity, sex and by socioeconomic position

A. Finnish Public Sector study				Sex						Socioeconomic position								
	All participants			Men			Women			High			Intermediate			Low		
Overall multimorbidity	WLE[1]	95% CI		WLE[2]	95% CI		WLE[2]	95% CI		WLE[3]	95% CI		WLE[3]	95% CI		WLE[3]	95% CI	
No disease	13.47	13.43	13.52	13.48	13.40	13.55	13.46	13.41	13.51	13.85	13.79	13.92	13.54	13.47	13.60	13.11	13.05	13.17
1 diasease	13.07	13.02	13.12	13.06	12.97	13.14	13.06	13.01	13.10	13.45	13.38	13.51	13.14	13.08	13.20	12.71	12.65	12.76
2 diseases	12.73	12.66	12.78	12.72	12.63	12.80	12.72	12.66	12.77	13.10	13.03	13.17	12.80	12.73	12.87	12.37	12.30	12.43
≥3 diseases	12.44	12.37	12.52	12.40	12.28	12.51	12.44	12.37	12.51	12.82	12.74	12.90	12.52	12.42	12.59	12.08	12.01	12.16

B. Health and Social Support study				Sex						Socioeconomic position								
	All participants			Men			Women			High			Intermediate			Low		
Overall multimorbidity	WLE[1]	95% CI		WLE[2]	95% CI		WLE[2]	95% CI		WLE[3]	95% CI		WLE[3]	95% CI		WLE[3]	95% CI	
No disease	10.58	10.43	10.72	10.41	10.24	10.59	10.70	10.52	10.86	12.20	11.94	12.39	10.67	10.51	10.82	9.76	9.57	9.95
1 diasease	9.80	9.63	9.93	9.61	9.43	9.78	9.92	9.75	10.07	11.47	11.23	11.68	9.89	9.71	10.05	8.97	8.78	9.15
2 diseases	9.04	8.84	9.22	8.81	8.57	9.05	9.18	8.97	9.38	10.75	10.44	11.02	9.14	8.93	9.35	8.17	7.93	8.37
≥3 diseases	7.72	7.50	7.94	7.52	7.25	7.76	7.86	7.61	8.13	9.51	9.17	9.81	7.82	7.55	8.07	6.85	6.56	7.09

Note: WLE: working life expectancy; SEP: socioeconomic position.

[1] Adjusted for sex and socioeconomic position. [2] Adjusted for socioeconomic position. [3] Adjusted for sex.

Supplementary table 4. Estimated working life expectancies from age 50 up to 68 years in Health and Social Support study, by multimorbidity, sex and socioeconomic position

	Men																	
	High SEP						Intermediate SEP						Low SEP					
	WLE	95% CI		Months lost	95% CI		WLE	95% CI		Months lost	95% CI		WLE	95% CI		Months lost	95% CI	
**Overall multimorbidity**																		
No disease	12.73	12.48	12.94	0	-	-	11.40	11.20	11.58	0	-	-	10.64	10.37	10.86	0	-	-
1 diasease	12.23	11.98	12.46	6.0	2.1	10.0	10.87	10.66	11.05	6.3	3.1	9.5	10.11	9.88	10.29	6.4	2.6	10.3
2 diseases	11.70	11.39	12.00	12.4	7.8	17.0	10.32	10.08	10.55	13.0	9.4	16.6	9.49	9.16	9.76	13.8	9.1	18.4
≥3 diseases	11.04	10.68	11.36	20.4	15.4	25.3	9.63	9.35	9.91	21.1	17.1	25.2	8.91	8.46	9.24	20.8	15.3	26.3
**Physical and mental multimorbidity**																		
≥2 physical diseases	11.52	11.21	11.78	14.6	10.2	19.0	10.12	9.84	10.33	15.4	11.7	19.0	9.31	9.02	9.56	16.0	11.6	20.3
≥1 physical diseases and depression	11.44	11.08	11.78	15.5	10.5	20.5	10.04	9.72	10.34	16.2	11.9	20.6	9.28	8.78	9.60	16.4	10.6	22.1

	Women																	
	High SEP						Intermediate SEP						Low SEP					
	WLE	95% CI		Months lost	95% CI		WLE	95% CI		Months lost	95% CI		WLE	95% CI		Months lost	95% CI	
**Overall multimorbidity**																		
No disease	12.77	12.53	13.00	0	-	-	11.43	11.24	11.59	0 (Ref)			10.78	10.59	10.98	0	-	-
1 diasease	12.27	12.02	12.49	6.0	2.1	10.0	10.90	10.72	11.09	6.3	3.3	9.4	10.25	10.04	10.44	6.4	3.1	9.7
2 diseases	11.77	11.50	12.06	12.0	7.7	16.3	10.39	10.17	10.63	12.5	9.1	16.0	9.72	9.49	9.96	12.8	9.1	16.4
≥3 diseases	11.08	10.73	11.40	20.3	15.4	25.2	9.68	9.42	9.92	21.0	17.4	24.7	9.03	8.71	9.29	21.0	16.8	25.2
**Physical and mental multimorbidity**																		
≥2 physical diseases	11.56	11.25	11.86	14.4	9.9	19.0	10.17	9.96	10.38	15.2	11.9	18.4	9.50	9.27	9.70	15.4	11.9	18.9
≥1 physical diseases and depression	11.47	11.13	11.80	15.5	10.7	20.4	10.08	9.83	10.35	16.3	12.5	20.0	9.42	9.11	9.67	16.4	12.3	20.4

Notes: WLE: working life expectancy; SEP: socioeconomic position. Months lost: months lost compared to no having no disease.

Analyses were based on data from 2 671 women and 2 122 men who worked at baseline.

Supplementary table 5. Estimated working life expectancies from age 50 up to 68 years in Finnish Public Sector study, by types of multimorbidity, sex and socioeconomic position

Men

	High SEP						Intermediate SEP						Low SEP					
	WLE	95% CI		Months lost	95% CI		WLE	95% CI		Months lost	95% CI		WLE	95% CI		Months lost	95% CI	
**Coronary heart disease or stroke and cardiovascular multimorbidity, %**																		
Coronary heart disease or stroke only	13.53	12.48	14.07	4.0	-5.6	13.6	13.22	11.89	13.77	3.7	-7.6	15.1	12.80	11.52	13.33	3.7	-7.2	14.7
Coronary heart disease or stroke with 1 other disease	13.03	12.60	13.38	10.1	5.3	14.9	12.72	12.23	13.08	9.7	4.4	14.9	12.31	11.80	12.63	9.6	4.6	14.7
Coronary heart disease or stroke with ≥2 other diseases	12.36	12.09	12.58	18.0	14.9	21.1	12.05	11.77	12.30	17.8	14.4	21.1	11.63	11.35	11.86	17.8	14.6	20.9
**Multimorbidity excluding coronary heart disease or stroke, %**																		
2 diseases without coronary heart disease or stroke	13.12	13.03	13.21	8.9	7.5	10.4	12.80	12.68	12.89	8.8	7.1	10.6	12.37	12.26	12.47	8.9	7.3	10.5
≥3 diseases without coronary heart disease or stroke	12.87	12.74	12.98	11.9	10.2	13.7	12.53	12.37	12.65	12.0	10.0	14.0	12.11	11.98	12.23	12.0	10.2	13.9
**Depression and physical-mental multimorbidity, %**																		
Depression only	13.45	13.25	13.63	4.9	2.4	7.4	13.11	12.88	13.30	5.1	2.3	7.9	12.68	12.46	12.89	5.2	2.4	8.0
Depression with 1 other disease	13.03	12.88	13.16	10.1	8.1	12.1	12.71	12.52	12.85	9.9	7.6	12.2	12.28	12.09	12.41	10.0	7.8	12.2
Depression with ≥2 other diseases	12.61	12.47	12.73	15.1	13.2	17.0	12.27	12.10	12.41	15.1	12.9	17.3	11.85	11.69	11.97	15.2	13.3	17.2
**Multimorbidity excluding depression, %**																		
2 diseases without depression	13.14	13.04	13.23	8.8	7.2	10.3	12.80	12.67	12.91	8.7	6.9	10.6	12.38	12.27	12.48	8.8	7.2	10.4
≥3 diseases without depresssion	12.99	12.84	13.13	10.5	8.5	12.5	12.64	12.48	12.79	10.7	8.4	12.9	12.22	12.05	12.37	10.7	8.6	12.8
**Cancer and malignant multimorbidity, %**																		
Cancer only	12.78	12.37	13.17	13.0	8.1	17.9	12.25	11.68	12.68	15.4	9.3	21.5	11.85	11.16	12.27	15.1	8.4	21.9
Cancer with 1 other disease	12.80	12.45	13.06	12.8	9.0	16.6	12.39	12.00	12.70	13.7	9.3	18.0	11.98	11.61	12.28	13.6	9.5	17.8
Cancer with ≥2 other diseases	12.69	12.40	12.94	14.1	10.7	17.5	12.31	11.90	12.57	14.7	10.5	18.9	11.89	11.58	12.17	14.7	11.0	18.3
**Multimorbidity excluding cancer, %**																		
2 diseases without cancer	13.14	13.04	13.23	8.7	7.2	10.3	12.82	12.71	12.91	8.6	6.9	10.3	12.39	12.29	12.49	8.7	7.2	10.2
≥3 diseases without cancer	12.83	12.71	12.94	12.5	10.7	14.2	12.49	12.35	12.62	12.5	10.5	14.4	12.07	11.93	12.19	12.6	10.7	14.4
**Diabetes and diabetic multimorbidity, %**																		
Diabetes only	13.39	12.84	13.77	5.7	0.0	11.4	13.11	12.54	13.50	5.1	-0.8	10.9	12.70	12.15	13.09	5.0	-0.8	10.8
Diabetes with 1 other disease	13.13	12.81	13.44	8.8	4.9	12.8	12.83	12.44	13.14	8.4	4.0	12.8	12.43	12.04	12.74	8.3	4.0	12.6
Diabetes with ≥2 other diseases	12.49	12.23	12.72	16.5	13.5	19.6	12.15	11.88	12.41	16.5	13.1	20.0	11.74	11.44	11.97	16.5	13.1	19.8
**Multimorbidity excluding diabetes, %**																		
2 diseases without diabetes	13.11	13.01	13.20	9.0	7.5	10.5	12.79	12.68	12.90	8.9	7.1	10.6	12.37	12.26	12.47	9.0	7.3	10.6
≥3 diseases without diabetes	12.87	12.75	12.98	12.0	10.2	13.7	12.54	12.40	12.66	11.9	10.0	13.9	12.11	11.99	12.24	12.0	10.3	13.8
**Musculoskeletal multimorbidity, %**																		
Musculoskeletal disorder only	13.45	13.34	13.54	5.0	3.5	6.6	13.13	13.03	13.23	4.8	3.1	6.4	12.71	12.61	12.81	4.9	3.3	6.4
Musculoskeletal disorder with 1 other disease	13.08	12.97	13.17	9.5	7.9	11.0	12.76	12.63	12.87	9.3	7.5	11.1	12.33	12.22	12.44	9.4	7.8	11.1
Musculoskeletal disorder with ≥2 other diseases	12.86	12.76	12.98	12.0	10.4	13.6	12.54	12.40	12.66	11.9	9.9	13.8	12.11	11.97	12.23	12.0	10.2	13.8
**Multimorbidity excluding musculoskeletal disorder, %**																		
2 diseases without msculoskeletal disorder	13.20	13.05	13.32	8.0	6.1	9.9	12.86	12.70	13.01	8.1	5.9	10.3	12.44	12.28	12.57	8.1	6.1	10.1
≥3 diseases without musculoskeletal disorder	12.52	12.24	12.73	16.2	13.0	19.3	12.09	11.72	12.33	17.3	13.4	21.1	11.67	11.31	11.95	17.4	13.5	21.3

Women

	High SEP						Intermediate SEP						Low SEP					
	WLE	95% CI		Months lost	95% CI		WLE	95% CI		Months lost	95% CI		WLE	95% CI		Months lost	95% CI	
**Coronary heart disease or stroke and cardiovascular multimorbidity, %**																		
Coronary heart disease or stroke only	13.48	12.71	13.94	4.3	-3.1	11.7	13.19	12.53	13.70	4.0	-3.0	11.1	12.77	11.87	13.24	4.0	-4.2	12.3
Coronary heart disease or stroke with 1 other disease	13.02	12.66	13.32	9.8	5.7	13.8	12.76	12.38	13.09	9.2	4.9	13.5	12.34	12.01	12.65	9.2	5.3	13.1
Coronary heart disease or stroke with ≥2 other diseases	12.35	12.14	12.55	17.9	15.3	20.5	12.07	11.84	12.28	17.5	14.8	20.2	11.64	11.41	11.84	17.5	14.9	20.2
**Multimorbidity excluding coronary heart disease or stroke, %**																		
2 diseases without coronary heart disease or stroke	13.09	13.01	13.16	9.0	7.8	10.2	12.79	12.71	12.87	8.9	7.7	10.0	12.36	12.29	12.43	8.9	7.8	10.1
≥3 diseases without coronary heart disease or stroke	12.87	12.79	12.96	11.6	10.4	12.8	12.57	12.48	12.65	11.5	10.3	12.8	12.14	12.06	12.22	11.6	10.4	12.8
**Depression and physical-mental multimorbidity, %**																		
Depression only	13.45	13.28	13.60	4.7	2.6	6.8	13.13	12.95	13.29	4.8	2.7	7.0	12.69	12.52	12.85	4.9	2.8	7.0
Depression with 1 other disease	12.97	12.84	13.08	10.5	8.8	12.1	12.67	12.53	12.79	10.3	8.6	12.0	12.23	12.09	12.35	10.5	8.8	12.2
Depression with ≥2 other diseases	12.61	12.50	12.73	14.7	13.1	16.3	12.31	12.20	12.42	14.6	13.2	16.1	11.87	11.76	11.99	14.7	13.2	16.3
**Multimorbidity excluding depression, %**																		
2 diseases without depression	13.13	13.05	13.21	8.5	7.3	9.7	12.83	12.75	12.91	8.4	7.2	9.6	12.39	12.32	12.46	8.5	7.4	9.6
≥3 diseases without depresssion	13.01	12.90	13.12	9.9	8.4	11.5	12.70	12.60	12.81	9.9	8.5	11.4	12.27	12.15	12.37	10.0	8.5	11.5
**Cancer and malignant multimorbidity, %**																		
Cancer only	13.20	12.91	13.48	7.7	4.2	11.2	12.80	12.47	13.08	8.7	5.0	12.4	12.39	12.06	12.65	8.6	4.9	12.2
Cancer with 1 other disease	12.92	12.68	13.14	11.1	8.2	13.9	12.58	12.33	12.83	11.4	8.4	14.5	12.15	11.90	12.37	11.5	8.6	14.4
Cancer with ≥2 other diseases	12.79	12.58	12.98	12.6	10.1	15.2	12.47	12.23	12.67	12.7	10.0	15.4	12.04	11.83	12.25	12.8	10.1	15.4
**Multimorbidity excluding cancer, %**																		
2 diseases without cancer	13.10	13.03	13.19	8.8	7.7	10.0	12.81	12.73	12.87	8.7	7.6	9.8	12.37	12.30	12.44	8.7	7.6	9.9
≥3 diseases without cancer	12.82	12.72	12.90	12.2	10.9	13.5	12.52	12.44	12.61	12.1	10.9	13.4	12.09	11.99	12.17	12.2	10.9	13.5
**Diabetes and diabetic multimorbidity, %**																		
Diabetes only	13.38	12.96	13.76	5.5	0.7	10.4	13.13	12.75	13.52	4.8	0.2	9.5	12.70	12.29	13.06	4.8	0.1	9.5
Diabetes with 1 other disease	13.18	12.92	13.44	7.9	4.7	11.1	12.93	12.66	13.17	7.2	4.0	10.3	12.51	12.23	12.76	7.1	3.8	10.3
Diabetes with ≥2 other diseases	12.56	12.37	12.75	15.3	12.9	17.6	12.29	12.07	12.47	14.9	12.4	17.4	11.86	11.67	12.07	14.9	12.3	17.4
**Multimorbidity excluding diabetes, %**																		
2 diseases without diabetes	13.08	13.02	13.15	9.1	8.0	10.2	12.79	12.71	12.85	8.9	7.8	10.0	12.35	12.28	12.41	9.0	7.9	10.1
≥3 diseases without diabetes	12.86	12.76	12.94	11.8	10.5	13.1	12.56	12.46	12.64	11.7	10.4	13.0	12.12	12.03	12.20	11.8	10.6	13.1
**Musculoskeletal multimorbidity, %**																		
Musculoskeletal disorder only	13.38	13.30	13.45	5.5	4.3	6.7	13.08	13.00	13.17	5.4	4.2	6.6	12.65	12.58	12.72	5.4	4.3	6.5
Musculoskeletal disorder with 1 other disease	13.04	12.95	13.12	9.6	8.4	10.9	12.74	12.65	12.82	9.5	8.2	10.8	12.31	12.23	12.38	9.5	8.4	10.7
Musculoskeletal disorder with ≥2 other diseases	12.84	12.76	12.93	12.0	10.7	13.2	12.55	12.46	12.64	11.8	10.5	13.0	12.12	12.04	12.19	11.8	10.6	13.0
**Multimorbidity excluding musculoskeletal disorder, %**																		
2 diseases without msculoskeletal disorder	13.20	13.08	13.31	7.7	6.2	9.2	12.90	12.78	13.00	7.6	6.1	9.1	12.47	12.36	12.58	7.6	6.1	9.1
≥3 diseases without musculoskeletal disorder	12.66	12.47	12.85	14.1	11.7	16.5	12.31	12.10	12.51	14.6	12.1	17.1	11.88	11.69	12.08	14.7	12.2	17.1

Notes: WLE: working life expectancy; SEP: socioeconomic position. Months lost: months lost compared to having no disease.

Supplementary table 6. Estimated working life expectancies from age 50 up to 68 years in Health and Social Support study,by types of multimorbidity, sex and socioeconomic position

	Men																	
	High SEP						Intermediate SEP						Low SEP					
Multimorbidity	WLE	95% CI		Months lost	95% CI		WLE	95% CI		Ionths los	95% CI		WLE	95% CI		Ionths los	95% CI	
**Coronary heart disease or stoke and cardiovascular multimorbidity, %**																		
Coronary heart disease or stroke only	12.21	10.14	13.37	-2.0	-21.6	17.6	10.73	9.10	11.85	-2.6	-19.3	14.0	9.67	6.47	10.85	-1.5	-27.9	24.9
Coronary heart disease or stroke with 1 other disease	9.34	5.05	10.01	32.5	2.6	62.4	7.69	5.86	8.31	33.9	19.1	48.8	6.73	3.37	7.37	33.8	9.6	57.9
Coronary heart disease or stroke with ≥2 other diseases	8.54	4.05	9.16	42.1	11.3	72.9	6.76	4.06	7.25	45.1	25.8	64.3	5.72	1.65	6.24	45.9	18.3	73.6
**Multimorbidity excluding coronary heart disease or stroke, %**																		
2 diseases without coronary heart disease or stroke	10.60	10.23	10.89	17.4	12.4	22.4	9.00	8.76	9.25	18.1	14.4	21.8	7.93	7.59	8.21	19.3	14.8	23.8
≥3 diseases without coronary heart disease or stroke	9.52	9.13	9.86	30.3	24.9	35.6	7.83	7.52	8.09	32.1	28.1	36.2	6.86	6.49	7.15	32.2	27.5	36.8
**Depression and physical-mental multimorbidity, %**																		
Depression only	10.05	9.31	10.53	24.0	16.1	31.9	8.38	7.87	8.85	25.6	19.4	31.9	7.39	6.53	7.85	25.9	17.6	34.3
Depression with 1 other disease	10.02	9.54	10.44	24.3	18.1	30.4	8.36	7.96	8.74	25.8	20.6	30.9	7.32	6.74	7.69	26.8	20.6	32.9
Depression with ≥2 other diseases	8.97	8.39	9.37	37.0	30.4	43.6	7.23	6.84	7.54	39.4	34.7	44.2	6.20	5.74	6.54	40.1	34.7	45.5
**Multimorbidity excluding depression, %**																		
2 diseases without depression	10.74	10.36	11.06	15.6	10.4	20.9	9.16	8.87	9.39	16.2	12.4	20.0	8.10	7.74	8.41	17.3	12.6	22.0
≥3 diseases without depresssion	9.77	9.25	10.18	27.3	20.9	33.6	8.11	7.64	8.46	28.8	23.5	34.2	7.11	6.30	7.45	29.2	21.9	36.6
**Cancer and malignant multimorbidity, %**																		
Cancer only	-	-	-				-	-	-				-	-	-			
Cancer with 1 other disease	-	-	-				-	-	-				-	-	-			
Cancer with ≥2 other diseases	-	-	-				-	-	-				-	-	-			
**Multimorbidity excluding cancer, %**	-	-	-				-	-	-				-	-	-			
2 diseases without cancer	-	-	-				-	-	-				-	-	-			
≥3 diseases without cancer	-	-	-				-	-	-				-	-	-			
**Diabetes and diabetic multimorbidity, %**																		
Diabetes only	10.09	2.74	10.88	23.5	-25.4	72.5	8.49	4.05	9.38	24.3	-7.8	56.3	7.51	1.22	8.31	24.5	-18.1	67.1
Diabetes with 1 other disease	9.79	8.31	10.52	27.1	13.5	40.7	8.13	7.32	8.78	28.5	19.5	37.5	7.17	4.83	7.85	28.5	10.1	46.8
Diabetes with ≥2 other diseases	8.26	7.10	8.82	45.5	34.7	56.2	6.51	5.73	7.05	48.0	39.7	56.2	5.51	3.77	6.08	48.5	34.4	62.5
**Multimorbidity excluding diabetes, %**																		
2 diseases without diabetes	10.58	10.23	10.89	17.6	12.6	22.6	9.01	8.75	9.24	18.0	14.3	21.7	7.94	7.64	8.21	19.2	15.0	23.5
≥3 diseases without diabetes	9.52	9.10	9.86	30.3	24.8	35.8	7.84	7.52	8.15	32.0	27.6	36.4	6.86	6.45	7.14	32.2	27.3	37.1
**Musculoskeletal multimorbidity, %**																		
Musculoskeletal disorder only	11.41	11.00	11.69	7.7	2.5	12.8	9.81	9.57	10.05	8.5	4.9	12.1	8.92	8.57	9.14	7.6	3.3	11.8
Musculoskeletal disorder with 1 other disease	10.50	10.13	10.82	18.6	13.4	23.7	8.90	8.59	9.15	19.3	15.3	23.3	7.82	7.45	8.11	20.7	16.0	25.4
Musculoskeletal disorder with ≥2 other diseases	9.26	8.87	9.61	33.4	28.0	38.9	7.55	7.20	7.81	35.5	31.2	39.7	6.55	6.22	6.83	36.0	31.5	40.4
**Multimorbidity excluding musculoskeletal disorder, %**																		
2 diseases without msculoskeletal disorder	10.66	10.28	11.03	16.6	11.1	22.1	9.00	8.60	9.33	18.1	13.3	23.0	8.06	7.62	8.38	17.9	12.6	23.1
≥3 diseases without musculoskeletal disorder	9.63	8.68	10.23	29.0	19.3	38.8	7.89	7.19	8.40	31.5	23.8	39.1	6.91	5.76	7.44	31.6	21.2	41.9

	Women																	
	High SEP						Intermediate SEP						Low SEP					
Multimorbidity	WLE	95% CI		Ionths los	95% CI		WLE	95% CI		Months lost	95% CI		WLE	95% CI		Months lost	95% CI	
**Coronary heart disease or stoke and cardiovascular multimorbidity, %**																		
Coronary heart disease or stroke only	12.49	11.38	13.58	-2.2	-15.7	11.2	10.99	9.63	12.21	-2.5	-18.1	13.1	10.11	8.72	11.23	-2.5	-17.8	12.8
Coronary heart disease or stroke with 1 other disease	9.62	7.89	10.32	32.2	17.3	47.0	7.98	7.19	8.68	33.6	24.4	42.8	7.10	5.87	7.74	33.6	22.2	45.1
Coronary heart disease or stroke with ≥2 other diseases	8.88	7.61	9.55	41.1	29.1	53.1	7.12	5.81	7.66	44.0	32.6	55.3	6.12	4.44	6.67	45.3	31.8	58.9
**Multimorbidity excluding coronary heart disease or stroke, %**																		
2 diseases without coronary heart disease or stroke	10.92	10.59	11.21	16.6	11.9	21.4	9.31	9.06	9.52	17.6	14.1	21.2	8.40	8.17	8.63	18.0	14.3	21.6
≥3 diseases without coronary heart disease or stroke	9.80	9.44	10.16	30.0	24.8	35.2	8.13	7.83	8.41	31.8	27.6	36.0	7.21	6.93	7.48	32.3	28.2	36.4
**Depression and physical-mental multimorbidity, %**																		
Depression only	10.36	9.85	10.86	23.3	16.6	30.0	8.71	8.27	9.22	24.8	18.7	31.0	7.78	7.27	8.24	25.4	19.1	31.8
Depression with 1 other disease	10.37	9.94	10.75	23.2	17.6	28.9	8.71	8.37	9.08	24.8	20.0	29.7	7.76	7.34	8.14	25.7	20.3	31.0
Depression with ≥2 other diseases	9.30	8.88	9.71	36.0	30.3	41.8	7.58	7.27	7.91	38.4	34.0	42.8	6.61	6.28	6.94	39.6	34.9	44.2
**Multimorbidity excluding depression, %**																		
2 diseases without depression	11.08	10.77	11.38	14.6	9.9	19.3	9.49	9.27	9.76	15.5	11.7	19.3	8.57	8.34	8.84	16.0	12.1	19.9
≥3 diseases without depresssion	10.09	9.64	10.53	26.6	20.5	32.7	8.44	8.07	8.83	28.2	23.1	33.2	7.50	7.11	7.84	28.9	23.9	33.9
**Cancer and malignant multimorbidity, %**																		
Cancer only	-	-	-				-	-	-				-	-	-			
Cancer with 1 other disease	-	-	-				-	-	-				-	-	-			
Cancer with ≥2 other diseases	-	-	-				-	-	-				-	-	-			
**Multimorbidity excluding cancer, %**	-	-	-				-	-	-				-	-	-			
2 diseases without cancer	-	-	-				-	-	-				-	-	-			
≥3 diseases without cancer	-	-	-				-	-	-				-	-	-			
**Diabetes and diabetic multimorbidity, %**																		
Diabetes only	10.38	6.31	11.19	23.1	-6.3	52.6	8.78	5.23	9.60	24.0	-2.3	50.3	7.89	4.12	8.71	24.2	-3.5	51.8
Diabetes with 1 other disease	10.12	9.28	10.80	26.2	16.6	35.8	8.49	7.69	9.16	27.5	18.4	36.7	7.60	6.61	8.28	27.7	17.4	38.0
Diabetes with ≥2 other diseases	8.59	7.97	9.20	44.6	36.7	52.5	6.86	6.31	7.46	47.1	39.8	54.3	5.89	5.25	6.45	48.1	40.5	55.7
**Multimorbidity excluding diabetes, %**																		
2 diseases without diabetes	10.91	10.63	11.19	16.7	12.3	21.1	9.32	9.10	9.57	17.5	13.9	21.2	8.41	8.19	8.62	17.9	14.4	21.5
≥3 diseases without diabetes	9.80	9.46	10.16	30.0	24.9	35.1	8.15	7.88	8.42	31.6	27.6	35.6	7.22	6.93	7.46	32.2	28.2	36.2
**Musculoskeletal multimorbidity, %**																		
Musculoskeletal disorder only	11.63	11.26	11.94	8.1	3.1	13.0	10.06	9.80	10.32	8.7	4.7	12.6	9.19	8.89	9.43	8.6	4.6	12.6
Musculoskeletal disorder with 1 other disease	10.84	10.52	11.13	17.6	12.9	22.3	9.24	8.96	9.52	18.6	14.5	22.6	8.30	8.03	8.55	19.3	15.3	23.3
Musculoskeletal disorder with ≥2 other diseases	9.57	9.23	9.92	32.7	27.7	37.8	7.89	7.64	8.18	34.7	30.7	38.7	6.94	6.66	7.19	35.6	31.6	39.6
**Multimorbidity excluding musculoskeletal disorder, %**																		
2 diseases without msculoskeletal disorder	10.95	10.55	11.27	16.2	11.0	21.4	9.33	8.98	9.68	17.4	12.6	22.2	8.43	8.08	8.69	17.7	13.3	22.2
≥3 diseases without musculoskeletal disorder	9.94	9.29	10.50	28.3	20.5	36.1	8.25	7.68	8.74	30.4	23.6	37.1	7.30	6.68	7.89	31.2	23.6	38.8

Notes: WLE: working life expectancy; SEP: socioeconomic position.

Supplementary table 7. Estimated working life expectancies from age 50 up to 68 years in Finnish Public Sector study and Health and Social Support study, by individual chronic diseases, senx and socioeconomic position

**A. Finnish Public Sector study**	**Men**									**Women**								
	**High SEP**			**Intermediate SEP**			**Low SEP**			**High SEP**			**Intermediate SEP**			**Low SEP**		
	**WLE**	**95% CI**		**WLE**	**95% CI**		**WLE**	**95% CI**		**WLE**	**95% CI**		**WLE**	**95% CI**		**WLE**	**95% CI**	
**Disease**																		
Respiratory disease	13.06	12.94	13.15	12.70	12.56	12.82	12.25	12.13	12.36	13.01	12.94	13.10	12.70	12.62	12.78	12.24	12.15	12.31
Hypertension	13.11	13.01	13.20	12.78	12.66	12.89	12.34	12.24	12.43	13.05	12.98	13.12	12.76	12.68	12.83	12.30	12.23	12.37
Coronary heart disease or stroke	12.72	12.52	12.89	12.40	12.16	12.60	11.95	11.69	12.15	12.64	12.47	12.81	12.36	12.20	12.53	11.91	11.74	12.06
Musculoskeletal disorder	13.16	13.07	13.24	12.84	12.75	12.93	12.42	12.33	12.51	13.09	13.04	13.15	12.79	12.73	12.85	12.37	12.32	12.43
Migraine	13.28	13.17	13.36	12.94	12.82	13.04	12.48	12.36	12.58	13.23	13.16	13.30	12.92	12.85	12.99	12.45	12.39	12.52
Depression	12.95	12.83	13.04	12.59	12.46	12.71	12.14	12.00	12.23	12.90	12.82	12.98	12.58	12.50	12.66	12.12	12.04	12.19
Diabetes	12.86	12.66	13.03	12.52	12.25	12.71	12.09	11.87	12.26	12.86	12.69	12.99	12.59	12.44	12.74	12.14	11.99	12.29
Cancer	12.79	12.57	12.99	12.32	12.04	12.54	11.89	11.62	12.11	12.95	12.81	13.08	12.58	12.40	12.72	12.13	11.98	12.26

**B. Health and Social Support study**	**Men**									**Women**								
	**High SEP**			**Intermediate SEP**			**Low SEP**			**High SEP**			**Intermediate SEP**			**Low SEP**		
	**WLE**	**95% CI**		**WLE**	**95% CI**		**WLE**	**95% CI**		**WLE**	**95% CI**		**WLE**	**95% CI**		**WLE**	**95% CI**	
**Disease**																		
Respiratory disease	10.34	10.00	10.64	8.59	8.32	8.82	7.53	7.21	7.79	10.53	10.25	10.84	8.77	8.53	9.00	7.77	7.52	7.98
Hypertension	10.46	10.08	10.74	8.69	8.43	8.93	7.62	7.33	7.88	10.56	10.22	10.90	8.80	8.55	9.03	7.81	7.55	8.04
Coronary heart disease or stroke	9.45	8.88	9.94	7.63	7.14	8.05	6.46	5.92	6.89	9.55	9.06	10.05	7.73	7.31	8.18	6.68	6.29	7.09
Musculoskeletal disorder	10.58	10.26	10.84	8.95	8.76	9.13	7.94	7.73	8.12	10.75	10.48	11.02	9.11	8.93	9.29	8.15	7.97	8.34
Migraine	11.06	10.70	11.35	9.34	9.07	9.56	8.31	7.98	8.56	11.22	10.91	11.51	9.50	9.28	9.68	8.51	8.29	8.73
Depression	9.77	9.41	10.05	7.93	7.66	8.19	6.83	6.50	7.06	9.98	9.67	10.27	8.16	7.93	8.37	7.14	6.87	7.36
Diabetes	9.33	8.75	9.84	7.50	7.06	7.83	6.39	5.87	6.76	9.47	9.01	9.90	7.65	7.20	8.05	6.64	6.25	7.02
Cancer	10.79	10.15	11.25	9.03	8.58	9.42	7.73	6.91	8.18	11.04	10.55	11.49	9.28	8.83	9.69	8.21	7.80	8.60

Notes: WLE: working life expectancy; SEP: socioeconomic position.
